# Advantage of using colonic washouts for Blastocystis detection in colorectal cancer patients

**DOI:** 10.1186/1756-3305-7-162

**Published:** 2014-04-03

**Authors:** Vinoth Kumarasamy, April Camilla Roslani, Kuppusamy Umah Rani, Suresh Kumar Govind

**Affiliations:** 1 Department of Parasitology, Faculty of Medicine, University of Malaya, Kuala Lumpur 50603, Malaysia; 2 Department of Surgery, Faculty of Medicine, University of Malaya, Kuala Lumpur 50603, Malaysia; 3 Department of Biomedical Science, Faculty of Medicine, University of Malaya, Kuala Lumpur 50603, Malaysia

**Keywords:** Colorectal cancer, Blastocystis, Subtypes

## Abstract

**Background:**

There have been previous studies associating microorganisms to cancer and with our recent findings of Blastocytsis antigen having a higher *in vitro* proliferation of cancer cells strengthens the suspicion. Collecting faecal samples alone to associate this parasite with cancer may not be accurate due to the phenomenon of irregular shedding and the possible treatment administrated to the cancer patients. Hence, this become the basis to search for an alternate method of sample collection. Colonic washout is an almost complete washed up material from colon and rectum which includes various microorganisms such as Blastocystis and other lodged material within the villi. The detection of parasite in colonic washouts will give a better reflection on the association between Blastocystis and CRC.

**Methods:**

Blastocytsis detection was made by *in vitro* culture method using Jones’ medium, formal ether concentration technique and conventional polymerase chain reaction (PCR) on faecal samples and colonic washouts of 204 CRC patients from colonoscopy procedure. Faecal samples and colonic washouts from 221 normal individuals served as control.

**Results:**

We observed an increased detection of Blastocystis using colonic washouts (n = 53, 12.47%) than faecal samples (n = 26, 6.12%). Eleven faecal samples showed positive results for Blastocystis which were also found in colonic washouts using the PCR technique. This study for the first time showed a significant Blastocystis infection among CRC patients (n = 43, 21.08%) compared to the asymptomatic normal individuals (n = 22, 9.95%). Blastocystis subtype 3 infection was found to be significantly more prevalent (n = 26, 12.75%) compared to other subtypes namely subtype 1: n = 9 (4.41%), subtype 2: n = 1 (0.49%), subtype 5: n = 1 (0.49%) and mixed subtype: n = 6 (2.94%) among the CRC patients.

**Conclusion:**

The study showed that colonic washouts provide a better alternative for Blastocystis detection in CRC patients compared to faecal samples as this prevents treatment regime and the phenomenon of irregular shedding from influencing the detection results obtained from faecal samples.

## Background

Blastocystis is one of the most commonly detected microorganisms in the human gut [1]. It is known to cause many non-specific symptoms such as stomach bloating and diarrhea [2]. Among colorectal cancer (CRC) patients, the prevalence of Blastocystis is yet to be determined probably due to the lack of evidence to show its pathogenic role.

Considering the fact that human intestine is often exposed to various microorganisms, the putative role of infectious agents in causing gastrointestinal disorders including CRC is undeniable. In addition, nearly 18% of all cancers worldwide were associated with infectious agents [3]. Human colon can easily allow the growth of over 500 different species of bacteria due to its environment which is rich in nutrients [4].

Blastocystis infection was also reported to be frequent in cancer and HIV/AIDS patients with gastrointestinal symptoms [5]. Although ten different subtypes of Blastocystis (Subtype 1 – Subtype 10) have been identified thus far [6-8], epidemiological studies related to Blastocystis have often been hampered by the poor sensitivity of standard methods available to detect Blastocystis genotypes in faecal samples. While a previous *in vitro* study have demonstrated that the solubilized antigen of Blastocystis could proliferate colon cancer cells [3], the present study attempts to assess the prevalence of Blastocystis in CRC patients. However, detection of Blastocystis becomes harder in this cohort as these patients are likely to undergo chemotherapy and other drug treatments that probably could have killed the organism. The irregular shedding reported previously [9] raises the possibility that Blastocystis detection in faecal samples could be missed. Therefore in the present study we attempted to compare the prevalence of Blastocystis using colonic washouts and faecal samples to identify the Blastocystis genotype present. It is highly probable that a better Blastocystis occurrence in patients with CRC can be obtained when colonic washouts are used as sample material. Patients resort to colonoscopy only as a last resort to finding the cause when the initial screening and treatment do not relieve symptoms. The possibility of re-covering parasites from colonic washout is higher as it contains an almost complete washed up material from colon and rectum which includes various microorganisms such as Blastocystis and other lodged material within villi.

## Methods

### Patients

This is a hospitalized-based cross-sectional study of 425 patients who underwent diagnostic colonoscopy in University of Malaya Medical Centre (UMMC). The samples were collected over a 2-year period, between 2010 and 2012. Colonoscopy is usually recommended for screening and prevention of colorectal cancer (CRC). Over 751 potential participants were briefed about this study but only 425 patients were interested. They were provided with a consent form in person. They belonged to two cohorts- a group of 221 patients who came for normal screening and 204 patients presenting colorectal malignancies. This study was approved by the Medical Ethics Committee of the UMMC in accordance with the declaration of Helsinki.

### Specimen collection and screening

Colonic washouts were obtained at the time of (or immediately prior to) the diagnosis of CRC. Whereas, faecal samples were obtained via the standard clinical procedures whereby patients will be given a duration of 1–2 weeks to deliver their faecal samples. Colonic washouts were collected in clean disposable bowls. Each sample was centrifuged at 1,400 × g and the pellets obtained were cultured in Jones’ medium [10] supplemented with 10% horse serum [11] and incubated at 37°C for 24 h and then screened for Blastocystis*.* DNA was extracted from the remaining fresh colonic washout for genotyping purposes. A pea size of the faecal samples collected from the same individuals were directly cultured in Jones’ medium and other procedures carried out were similar to that of colonic washout. Predominant Blastocystis subtype was identified using polymerase chain reaction (PCR) technique.

### Formal ether concentration technique

Fresh colonic washout and faecal samples were routinely processed by the formal ether concentration technique (FECT) to obtain stool concentrate. Ficoll-Paque density gradient centrifugation method was carried out to isolate Blastocystis cyst from the concentrate [11]. Briefly, the stool concentrate re-suspended in PBS was layered on 5 ml of Ficoll-Paque and centrifuged at 1,600 × g for 20 minutes. Blastocystis cyst layer which was formed after centrifugation was removed into another Falcon tube and re-suspended in 1 ml PBS and observed under microscope for the detection of Blastocystis cyst.

### DNA extraction and genotyping

PCR technique was used to detect Blastocystis in addition to standard stool culture technique in both colonic washouts and faecal samples. DNA extraction was conducted usingQIAamp® DNA Stool Mini Kit (QIAGEN). Briefly, 200 ml of the sediments of colonic washout samples or faecal samples were used to extract DNA. PCR was carried out with specific sequence-tagged site (STS) primers (Table 1). Seven different STS primers were used for genotyping as described previously [6,7,12-18]. The STS primers used were SB83 (351 bp), SB340 (704 bp), SB227 (526 bp), SB337 (487 bp), SB336 (317 bp), SB332 (338 bp) and SB 155 (650 bp) for subtype 1, 2, 3, 4, 5, 6 and 7 respectively based on a recent classification terminology [19]. One microliter of DNA preparation was used to amplify the genomic sequences in a 20 μl PCR cocktail containing 0.2 mM of the four dNTPs, 25 pmol of each primer, 1× PCR buffer (75 mMTris–HCl, pH 8.8, 20 mM (NH_4_)_2_SO_4_, and 0.01% Tween 20), 2.5 mM MgCl, and 1 U Taq DNA polymerase (recombinant) (Fermentas). PCR was carried out with one cycle denaturing at 95°C for 5 min, 42 cycles including annealing at 56.3°C for 90 s, extending at for 60 s, and additional cycle with a 10 min chain elongation at 72°C (Thermal cycler, BIO-RAD, USA). The PCR products and a size marker of a 100-bp ladder were electrophoresed in 1.5% agarose gels (Promega, USA) which were stained with ethidium bromide and photographed using an ultraviolet gel documentation system (Uvitec, UK). PCR amplification for each primer pair was repeated thrice for each positive sample.

**Table 1:** Primer sequences used for Blastocystis genotyping

**Subtypes**	**STS primer sets**	**Product size (bp)**	**Sequences of forward ( *F *) and reverse ( *R *) primers (5′-3′)**	**Source of primer**	**GenBank accession no.**	**Reference**	**Clade in the SSU rRNA phylogeny**^**a**^
1	SB83	351	F GAAGGACTCTCTGACGATGA	Nand II	AF166086	Yoshikawa et al. [6]	I
			R GTCCAAATGAAAGGCAGC				
2	SB155	650	F ATCAGCCTACAATCTCCTC	B	AF166087		VII
			R ATCGCCACTTCTCCAAT				
3	SB227	526	F TAGGATTTGGTGTTTGGAGA R	HV93–13	AF166088	Yoshikawa et al. [7]	III
			TTAGAAGTGAAGGAGATGGAAG				
4	SB332	338	F GCATCCAGACTACTATCAACATT	HJ96AS-1	AF166091		VI
			R CCATTTTCAGACAACCACTTA				
5	SB340	704	F TGTTCTTGTGTCTTCTCAGCTC	HJ96–1	AY048752	Yoshikawa et al. [18]	II
			R TTCTTTCACACTCCCGTCAT				
6	SB336	317	F GTGGGTAGAGGAAGGAAAACA	SY94–3	AY048751		V
			R AGAACAAGTCGATGAAGTGAGAT				
7	SB337	487	F GTCTTTCCCTGTCTATTCTGCA	RN94–9	AY048750		IV
			R AATTCGGTCTGCTTCTTCTG				

### Statistical analysis

Data were analyzed using Statistical Package for Social Sciences for Windows SPSS (Version 17.0). The Chi squared test was used to determine significance of differences in prevalence of Blastocystis between the healthy individuals and CRC patients. Fisher’s Exact test was used to determine the pre-dominant Blastocystis subtype in the colonic washouts of normal and CRC patients. In all the analyses, a probability level of *p* < 0.05 was used to indicate statistical significance.

## Results

### Blastocystis detection in the faecal samples and colonic washouts

The overall prevalence of Blastocystis infection obtained from the three methods used was 15.29% (65/425). There were 65/425, 4/425, and 4/425 samples (including faecal samples and colonic washouts) detected positive for Blastocystis via PCR, *in vitro* cultivation and formal ether concentration technique respectively. Overlapping positive results were often observed in faecal samples and colonic washouts as well as among the different techniques used. Colonic washouts and faecal samples showed 12.24% (n = 52) and 5.65% (n = 24) of Blastocystis infection respectively via the conventional PCR technique. Forty-one colonic washouts were positive for Blastocystis*,* despite the faecal samples from the same patients being negative for Blastocystis. Whereas, 11 faecal samples identified positive for Blastocystis also showed positive for colonic washouts obtained from the same patients using PCR technique. A very small percentage (0.96%, n = 4) of faecal samples were found positive via *in vitro* cultivation of faecal samples but none from colonic washouts. Although other parasites such as *Ascaris lumbricoides* and hook worm were detected via formal ether concentration technique but the frequency was negligible and statistically non-significant.

### Blastocystis infection and subtype analysis in CRC patients

**Figure 1:**
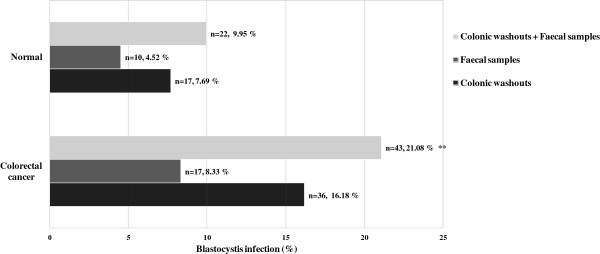
**Percentage of Blastocystis infection in colonic washouts and faecal samples collected from CRC and normal individuals.** **p < 0.01 is the comparison done between CRC patients and normal individuals.

A total of 43 (21.08%) samples were positive for Blastocystis infection in CRC patients and was significantly higher compared to normal individuals (n = 22, 9.95%, *p* < 0.01) (Figure 1). We conducted conventional PCR [20] to classify Blastocystis into certain subtype. In the current study, four different Blastocystis genotypes were identified among all the subjects namely subtype 1 (ST1), subtype 2 (ST2), subtype 3 (ST3), subtype 5 (ST5) and mixed subtypes. Subtype 6 (ST6) and subtype 7 (ST7) were not detected in all cases. ST3 was present at higher levels compared to other subtypes detected in both groups as shown in Table 2. Overall, ST3 was the most prevalent subtype (n = 30, 14.71%), whereas ST1, ST2, and ST5 were seen in 5.39% (n = 11), 3.43% (n = 7) and 0.49% (n = 1) of the CRC patients, respectively. Besides that, mixed subtype infections were detected in six samples which were 0.98% (n = 2, ST1 and ST2) and 1.96% (n = 4, ST2 and ST3) (Table 2). ST3 infection was also found to be statistically significant in CRC patients as compared with the control group (Table 2).

**Table 2:** Blastocystis genotypes found in CRC patients and normal individuals

**Blastocystis subtype**	**Colorectal cancer patients (n = 204)% (No. positive)**	**Healthy individuals (n = 221)% (No. positive)**
Subtype 1	4.41 (9)*	2.71 (6)
Subtype 2	0.49 (1)**	0.90 (2)*
Subtype 3	12.75 (26)^#^	3.17 (7)
Subtype 1 + 2	0.98 (2)**	0 (0)
Subtype 2 + 3	1.96 (4)*	1.81 (4)
Subtype 5	0.49 (1)**	1.36 (3)

*p < 0.05; **p < 0.01 are levels of significance between subtype 3 *Blastocystis* and other subtypes.

^#^p < 0.05 is the significant difference in *Blastocystis* (subtype) infection between CRC patients and healthy individuals.

## Discussion

Faecal sample collection of more than three times from the same person have been shown to significantly raise the possibility of detecting parasites [9]. However this would be too tedious and troublesome to execute. The faecal sample collection was purposely carried out as how it would be for routine screening purpose after the CRC diagnosis regardless of pre or post-treatment regime which could not be avoided. Therefore, the chances of recovering parasites which could have been influenced by the treatment regime cannot be overruled. The irregular shedding of Blastocystis in faecal sample further compound the challenge for detecting these parasites despite the use of the standard faecal culture technique [9]. In addition, detection using microscope is a greater challenge when parasites present in very small numbers in the faecal samples. As such, the usage of colonic washout probably far more effective as it can be collected almost immediately during diagnosis and the detection without the influence of any previous treatment regime. The diagnostic method to determine the presence of Blastocystis vary widely in sensitivity. Possibility of Blastocystis being eliminated by treatment usually sought by CRC patients for their initial symptoms could have resulted in zero detection in the present study using both formal-ether concentration technique and the gold standard *in vitro* cultivation method. Therefore, PCR technique was employed for the screening of Blastocystis in both faecal samples and colonic washouts. Blastocystis was detected in colonic washout samples from patients who were tested negative for Blastocystis by using faecal sample. This is the first study that detects Blastocystis in colonic washout samples from CRC patients via the conventional PCR method. These samples were collected at the initial stage of cancer detection which excluded the possibility of the patients undergoing medical treatments such as chemotherapy which could have killed the parasites. Although pathogenicity of Blastocystis is controversial, Blastocystis screening in CRC patients is crucial considering the recent *in vitro* studies providing evidences of the parasite’s exacerbating potential in proliferating cancer cells [21-23]. This method probably can be one of the more effective ways to screen for Blastocystis for high risk individuals and those who are suspected of infection but found negative by other less sensitive methods. The collection of colonic washouts is easier as it is a waste product produced from colonoscopy procedure. Furthermore, the chances for cross-contamination can be prevented as it can be collected directly and almost immediately from patients who are undergoing colonoscopy. This method may preferably be used for individuals that go through colonoscopy procedure for other diagnosis purposes such as CRC and those who are suspected of infection but found to be negative by other available methods.

Predominance of Blastocystis ST3 was similar to a previous study conducted among patients in a hospital in Singapore [24]. Blastocystis ST3 was reported to be the only subtype of human origin, while the rest being zoonotic [25]. As an earlier report suggested a possible correlation between ST3 and pathogenic potential [26], it is crucial for subtype identification. This will enable us to understand better, the possible pathogenic role of these subtypes play to the worsening of cancer. Furthermore, the occurrence of other cases such as acute urticaria and gastrointestinal symptoms were reported in patients with ST3 infection [27]. We observed a very low prevalence of ST1 and ST2 in both CRC and healthy individuals. These subtypes were mainly associated with zoonotic transmission. Similarly, ST4, ST6 and ST7 were not found in these patient groups as they are mostly zoonotic microorganisms [28].

## Conclusion

In conclusion, this study demonstrated that colonic washouts can be a better alternative to fecal samples to examine for Blastocystis infection especially in CRC cases. Our study shows that Blastocystis infection is common in CRC patients and it indicates subtype 3 as predominant among these individuals. However, the pathogenic role of this parasite in CRC patients is still unclear. Therefore, further study has to be conducted to determine the correlation between the genotype and symptomatology.

## Competing interests

The authors declare that they have no competing interest.

## Authors’ contributions

VK, SKG, ACR and URK participated in the design of the study. VK did the data and sample collections from the patients. VK, SKG, ACR and URK did the data analysis. VK wrote the manuscript. All authors read and approved the final manuscript.

## References

[B1] Zierdt C Rude W Bull B Protozoan characteristics of *Blastocystis hominis* Am J Clin Pathol 1967 48 495 6058384 10.1093/ajcp/48.5.495

[B2] Suresh K Venilla G Tan T Rohela M In vivo encystation of *Blastocystis hominis* Parasitol Res 2009 104 1373 1380 10.1007/s00436-009-1340-1 19238443

[B3] Parkin DM The global health burden of infection‒associated cancers in the year 2002 Int J Cancer 2006 118 3030 3044 10.1002/ijc.21731 16404738

[B4] Guarner F Enteric flora in health and disease Digestion 2006 73 5 12 10.1159/000089775 16498248

[B5] Tan T Ong S Suresh K Genetic variability of *Blastocystis* sp. isolates obtained from cancer and HIV/AIDS patients Parasitol Res 2009 105 1283 1286 10.1007/s00436-009-1551-5 19603182

[B6] Yoshikawa H Nagano I Wu Z Yap EH Singh M Takahashi Y Genomic polymorphism among *Blastocystis hominis* strains and development of subtype-specific diagnostic primers Mol Cell Probes 1998 12 153 159 10.1006/mcpr.1998.0161 9664577

[B7] Yoshikawa H Abe N Iwasawa M Kitano S Nagano I Wu Z Takahashi Y Genomic analysis of *Blastocystis hominis*Strains isolated from two long-term health care facilities J Clin Microbiol 2000 38 1324 1330 10747102 10.1128/jcm.38.4.1324-1330.2000 PMC86440

[B8] Yoshikawa H Abe N Wu Z PCR-based identification of zoonotic isolates of *Blastocystis* from mammals and birds Microbiology 2004 150 1147 1151 10.1099/mic.0.26899-0 15133074

[B9] Vennila G Suresh Kumar G Khairul Anuar A Rajah S Saminathan R Sivanandan S Ramakrishnan K Irregular shedding of *Blastocystis hominis* Parasitol Res 1999 85 162 164 10.1007/s004360050528 9934969

[B10] Jones W The experimental infection of rats with Entamoeba histolytica; with a method for evaluating the anti-amoebic properties of new compounds Ann Trop Med Parasitol 1946 40 130 20997895 10.1080/00034983.1946.11685270

[B11] Suresh K Smith H Comparison of methods for detecting *Blastocystis hominis* Eur J Clin Microbiol Infect Dis 2004 23 509 511 10.1007/s10096-004-1123-7 15168139

[B12] Abe N Wu Z Yoshikawa H Molecular characterization of *Blastocystis* isolates from birds by PCR with diagnostic primers and restriction fragment length polymorphism analysis of the small subunit ribosomal RNA gene Parasitol Res 2003 89 393 396 12632154 10.1007/s00436-002-0782-5

[B13] Abe N Wu Z Yoshikawa H Molecular characterization of *Blastocystis* isolates from primates Vet Parasitol 2003 113 321 325 10.1016/S0304-4017(03)00081-5 12719144

[B14] Abe N Wu Z Yoshikawa H Zoonotic genotypes of *Blastocystis hominis* detected in cattle and pigs by PCR with diagnostic primers and restriction fragment length polymorphism analysis of the small subunit ribosomal RNA gene Parasitol Res 2003 90 124 128 12756546 10.1007/s00436-002-0821-2

[B15] Li LH Zhang XP Lv S Zhang L Yoshikawa H Wu Z Steinmann P Utzinger J Tong XM Chen SH Cross-sectional surveys and subtype classification of human *Blastocystis* isolates from four epidemiological settings in China Parasitol Res 2007 102 83 90 10.1007/s00436-007-0727-0 17912552

[B16] Li L-H Zhou X-N Du Z-W Wang X-Z Wang L-B Jiang J-Y Yoshikawa H Steinmann P Utzinger J Wu Z Molecular epidemiology of human *Blastocystis* in a village in Yunnan province, China Parasitol Int 2007 56 281 286 10.1016/j.parint.2007.06.001 17627869

[B17] Yan Y Su S Lai R Liao H Ye J Li X Luo X Chen G Genetic variability of *Blastocystis hominis* isolates in China Parasitol Res 2006 99 597 601 10.1007/s00436-006-0186-z 16688468

[B18] Yoshikawa H Abe N WU Z Genomic polymorphism among *Blastocystis* isolates and development of PCR-based identification of zoonotic isolates J Eukaryot Microbiol 2003 50 710 711 10.1111/j.1550-7408.2003.tb00698.x 14736230

[B19] Stensvold CR Suresh GK Tan KS Thompson R Traub RJ Viscogliosi E Yoshikawa H Clark CG Terminology for *Blastocystis* subtypes–a consensus Trends Parasitol 2007 23 93 96 10.1016/j.pt.2007.01.004 17241816

[B20] Stensvold C Traub R von Samson-Himmelstjerna G Jespersgaard C Nielsen H Thompson R *Blastocystis*: Subtyping isolates using pyrosequencing™ technology Exp Parasitol 2007 116 111 119 10.1016/j.exppara.2006.12.002 17266951

[B21] Chandramathi S Suresh K Kuppusamy UR Solubilized antigen of *Blastocystis hominis* facilitates the growth of human colorectal cancer cells, HCT116 Parasitol Res 2010 106 941 945 10.1007/s00436-010-1764-7 20165878

[B22] Chan KH Chandramathi S Suresh K Chua KH Kuppusamy UR Effects of symptomatic and asymptomatic isolates of *Blastocystis hominis* on colorectal cancer cell line, HCT116 Parasitol Res 2012 110 2475 2480 10.1007/s00436-011-2788-3 22278727

[B23] Kumarasamy V Kuppusamy UR Samudi C Kumar S *Blastocystis* sp. subtype 3 triggers higher proliferation of human colorectal cancer cells, HCT116 Parasitol Res 2013 110 3551 3555 23933809 10.1007/s00436-013-3538-5

[B24] Wong KHS Ng G Lin RTP Yoshikawa H Taylor MB Tan KSW Predominance of subtype 3 among *Blastocystis* isolates from a major hospital in Singapore Parasitol Res 2008 102 663 670 10.1007/s00436-007-0808-0 18064490

[B25] Noël C Dufernez F Gerbod D Edgcomb VP Delgado-Viscogliosi P Ho LC Singh M Wintjens R Sogin ML Capron M Molecular phylogenies of *Blastocystis* isolates from different hosts: implications for genetic diversity, identification of species, and zoonosis J Clin Microbiol 2005 43 348 355 10.1128/JCM.43.1.348-355.2005 15634993 PMC540115

[B26] Tan T Suresh K Smith H Phenotypic and genotypic characterisation of *Blastocystis hominis* isolates implicates subtype 3 as a subtype with pathogenic potential Parasitol Res 2008 104 85 93 10.1007/s00436-008-1163-5 18795333

[B27] Katsarou-Katsari A Vassalos CM Tzanetou K Spanakos G Papadopoulou C Vakalis N Acute urticaria associated with amoeboid forms of *Blastocystis* sp. subtype 3 Acta Dermatovenereol Stockholm 2008 88 80 10.2340/00015555-0338 18176765

[B28] Boorom KF Smith H Nimri L Viscogliosi E Spanakos G Parkar U Li LH Zhou XN Ok U Leelayoova S Oh my aching gut: irritable bowel syndrome, Blastocystis, and asymptomatic infection Parasit Vectors 2008 1 40 10.1186/1756-3305-1-40 18937874 PMC2627840

